# Safety, Tolerability, and Immunogenicity of Measles and Rubella Vaccine Delivered with a High-Density Microarray Patch: Results from a Randomized, Partially Double-Blinded, Placebo-Controlled Phase I Clinical Trial

**DOI:** 10.3390/vaccines11111725

**Published:** 2023-11-17

**Authors:** Ben Baker, Imogen M. Bermingham, Indika Leelasena, Julian Hickling, Paul R. Young, David A. Muller, Angus H. Forster

**Affiliations:** 1 Vaxxas Pty Ltd., Hamilton, QLD 4007, Australia; bbaker@vaxxas.com (B.B.);; 2 University of the Sunshine Coast Clinical Trials Centre, Sippy Downs, QLD 4556, Australia; 3 Working in Tandem Ltd., Cambridge CB1 7AB, UK; 4 School of Chemistry and Molecular Biosciences, University of Queensland, St. Lucia, QLD 4072, Australia

**Keywords:** microarray patch (MAP), high-density microarray patch (HD-MAP), measles (M), rubella (R), vaccine, Phase I, clinical trial, thermostability

## Abstract

Microarray patches (MAPs) have the potential to be a safer, more acceptable, easier-to-use, and more cost-effective means for the administration of vaccines than injection by needle and syringe. Here, we report findings from a randomized, partially double-blinded, placebo-controlled Phase I trial using the Vaxxas high-density MAP (HD-MAP) to deliver a measles rubella (MR) vaccine. Healthy adults (N = 63, age 18–50 years) were randomly assigned 1:1:1:1 to four groups: uncoated (placebo) HD-MAPs, low-dose MR HD-MAPs (~3100 median cell-culture infectious dose [CCID_50_] measles, ~4300 CCID_50_ rubella); high-dose MR-HD-MAPs (~9300 CCID_50_ measles, ~12,900 CCID_50_ rubella); or a sub-cutaneous (SC) injection of an approved MR vaccine, MR-Vac (≥1000 CCID_50_ per virus). The MR vaccines were stable and remained viable on HD-MAPs when stored at 2–8 °C for at least 24 months. When MR HD-MAPs stored at 2–8 °C for 24 months were transferred to 40 °C for 3 days in a controlled temperature excursion, loss of potency was minimal, and MR HD-MAPs still met World Health Organisation (WHO) specifications. MR HD-MAP vaccination was safe and well-tolerated; any systemic or local adverse events (AEs) were mild or moderate. Similar levels of binding and neutralizing antibodies to measles and rubella were induced by low-dose and high-dose MR HD-MAPs and MR-Vac. The neutralizing antibody seroconversion rates on day 28 after vaccination for the low-dose HD-MAP, high-dose HD-MAP and MR-Vac groups were 37.5%, 18.8% and 35.7%, respectively, for measles, and 37.5%, 25.0% and 35.7%, respectively, for rubella. Most participants were seropositive for measles and rubella antibodies at baseline, which appeared to negatively impact the number of participants that seroconverted to vaccines delivered by either route. The data reported here suggest HD-MAPs could be a valuable means for delivering MR-vaccine to hard-to-reach populations and support further development. Clinical trial registry number: ACTRN12621000820808.

## 1. Introduction

In 2012, the Measles and Rubella Initiative (M&RI) set the ambitious goal of achieving measles (M) and rubella (R) elimination in at least five World Health Organisation (WHO) regions by 2020 [1]. By the end of 2015, elimination of rubella had been achieved in 55 countries [1], and by 2019 measles elimination had been achieved in 83 countries [2]. Despite this progress, estimated global coverage with the first dose of measles-containing vaccines (MCV1) has remained at 84–85% since 2010 [2]. This is far short of the ≥95% coverage with two doses of MCVs required for elimination [2], and means that almost one fifth of the global birth cohort is not vaccinated against measles. Furthermore, the COVID-19 pandemic has caused a significant drop in immunization services and coverage with MCV1 vaccines [3]. A midterm review and a research prioritization exercise by the MR&I identified microarray patches (MAPs) delivering MR vaccines as a potential game-changer for improving coverage, but also recognized that significant questions needed to be addressed if they were to fulfil their potential [4,5].

MAPs consist of arrays of hundreds-to-thousands of micro-projections, each <1 mm in length, which are coated with, or formed from, vaccine plus stabilizing excipients. Several formats have been evaluated in Phase I clinical trials with vaccines [6,7,8,9,10], and have been shown to be safe and well tolerated, and have induced immune responses that are similar to [6,7,8], or greater than [9,10] those seen with conventional injection of the same vaccine. Preclinical studies have shown that MAPs delivering MR vaccine are immunogenic and protect against measles challenge in infant rhesus macaques [11]. In addition to potential dose-sparing, MAPs offer several practical advantages compared with injection by needle and syringe (N&S), including improved thermostability, ease of use, greater acceptability by healthcare workers and recipients, avoidance of needle-stick injuries, and avoidance of reconstitution [12,13].

**Figure 1:**
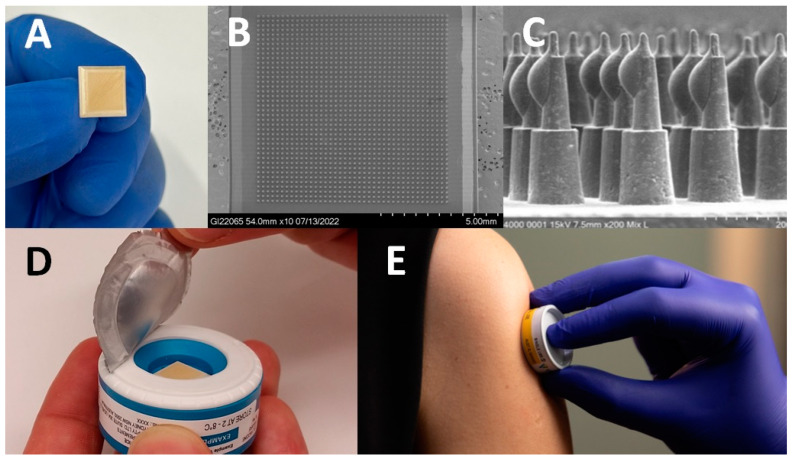
Overview of Vaxxas high-density microarray patch (HD-MAP) technology. (**A**) The ~1 cm^2^ HD-MAP. (**B**) Scanning electron micrograph of the array of ~1600 projections on the HD-MAP. (**C**) Scanning electron micrograph of vaccine-coated projections on the HD-MAP before application to a subject. (**D**) The HD-MAP is protected by a foil seal over the skin-facing opening of the applicator device and the foil seal is removed immediately before application. (**E**) The HD-MAP was applied to the upper arm of all participants in the HD-MAP groups.

The Vaxxas high-density MAP (HD-MAP), shown in Figure 1, differs from other MAPs in that it has a higher-density array of solid micro-projections (thousands per cm^2^) formed from medical-grade polymer, and vaccine antigens are dispensed onto the tips of the projections and dried. In the appropriate formulation, vaccines, including MR, coated onto HD-MAPs have improved thermostability compared with standard formulations [10,14,15]. A previous Vaxxas study demonstrated the HD-MAP could deliver vaccine dose-sparing by producing equivalent immune responses to an intramuscular injection using one-sixth of the dose of a monovalent influenza vaccine [10]. The vaccine-coated microarray is contained in an integrated, single-use, auto-disabling applicator that contains a spring that applies the HD-MAP to the skin at the correct kinetic energy required for micro-projection penetration.

Here we report the first Phase I trial of the Vaxxas HD-MAP used to deliver a MR vaccine. This is also the first clinical study with the HD-MAP and integrated applicator similar to that envisaged for commercial use.

## 2. Materials and Methods

### 2.1. Trial Participants and Study Design

The study was approved by the Bellberry Human Research Ethics Committee (South Australia), and conducted in accordance with the Australian National Health and Medical Research Council’s National Statement of Ethical Conduct in Human Research (2007, incorporating all updates as of May 2015), application number 2021-01-038. Written informed consent was obtained from all participants. The trial was registered with the Australian New Zealand Clinical Trials Registry (ANZCTR.org.au, accessed on 28 June 2021), trial ID ACTRN12621000820808.

The study was a randomized, partially double-blinded, placebo-controlled trial conducted at the University of the Sunshine Coast Clinical Trial Centre (QLD, Australia). Clinical staff and participants were blind as to which HD-MAP treatment was administered. All laboratory investigators were blind to treatment and participant allocation. The primary objective was to measure the safety and tolerability of MR vaccines delivered by HD-MAP in comparison to an uncoated HD-MAP and SC injection of a WHO prequalified MR vaccine (MR-Vac, Serum Institute of India Ltd., Pune, India). Exploratory outcomes were to evaluate the immune responses to HD-MAP application by foci-reducing neutralization titre (FRNT) and IgG ELISA.

**Figure 2:**
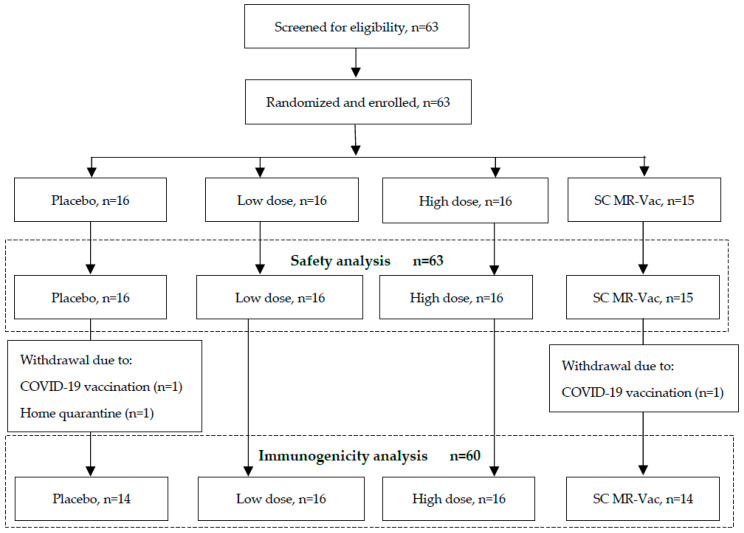
Trial profile.

Healthy males and females (non-pregnant and non-nursing) aged 18–50 years, with a BMI in the range of 18–32 kg/m^2^ (*n* = 63), were recruited and randomly allocated into one of four vaccination groups with ≥15 participants per group. Randomization was pre-determined, and sealed participant-specific code break envelopes were produced by the statistician responsible for preparing the randomization. The randomization was provided to the unblinded pharmacist for re-labelling of the investigational products. The four treatment groups were: uncoated (placebo) HD-MAPs; low-dose HD-MAP (~3100 and ~4300 median cell culture infectious dose (CCID_50_) measles and rubella, respectively); high-dose HD-MAP (~9300 and ~12,900 CCID_50_ measles and rubella, respectively); SC control (≥1000 CCID_50_ per virus). MR Vac tested during the study was determined to contain 1300 and 5200 CCID_50_ measles and rubella per dose. Neither HD-MAP group was intended to dose-match the SC control group. The group size was not based on any formal statistical calculations, as is typically the case for Phase I vaccination studies. However, the 15 participants in a group would have an 80% probability of showing at least one adverse event if the true rate of that event was more than 10.2%, and over the 45 participants receiving any MAP there was an 80% probability of showing at least one adverse event if the true rate of that event was more than 3.6%. The demographic profile of the participants is provided in Table 1, and the study disposition is shown in Figure 2.

### 2.2. Vaccines

Clarified virus pools for both M and R were supplied by Serum Institute of India Pvt. Ltd. (Pune, India) and processed at Vaxxas. The clarified pools were combined to produce a combined MR bulk which was concentrated by tangential flow filtration so that the required dose could be loaded onto HD-MAPs, and to exchange the harvest buffer for one containing excipients (sorbitol, L-histidine, trehalose dihydrate, sodium phosphate dibasic dihydrate, sodium phosphate monobasic dihydrate, water for injection and hydrolyzed porcine gelatin) to stabilize the M and R vaccines on HD-MAPs [15]. MR-Vac (Batch 0090N001B, expiry June 2022, supplied by Serum Institute of India) was used as SC control. The potency of MR-Vac was determined to be 1300 CCID_50_ measles per dose and 5200 CCID_50_ rubella per dose.

### 2.3. HD-MAP Manufacture

HD-MAPs were manufactured by injection moulding of a polymer, to produce HD-MAPs of 10.6 × 10.6 mm with approximately 1600 projections per patch. Each projection was approximately 350 µm high and 120 µm wide at the base. Vaccine was aseptically applied to the tips of each projection of gamma-irradiated (≥25 kGy, Steritech, Australia) HD-MAPs using the ‘M-jet’ process developed by Vaxxas. HD-MAPs were produced to deliver a single dose-level of ≥1000 CCID_50_ of each virus (M and R) per HD-MAP. The doses cited throughout this report refer to the estimated delivered dose. Preparatory studies using ex vivo and in vivo porcine skin assays determined the delivery of this MAP-vaccine combination to be approximately 50% of the coated dose; therefore, to deliver the desired dose, ≥2000 CCID_50_ of both M and R were loaded onto each HD-MAP. After HD-MAP manufacture, the per-MAP coated doses were measured at 6200 and 8600 CCID_50_ for M and R, respectively. The delivered doses were estimated to be 3100 and 4300 CCID_50_ per MAP for M and R, respectively. After coating with MR vaccine, the HD-MAPs were contained within an integrated applicator containing a dome spring, with a foil seal covering the skin-facing side of the applicator and packed in a foil pouch.

### 2.4. Vaccination Procedure

Three HD-MAPs were applied to all HD-MAP recipients. The high dose was achieved by applying three MR-coated HD-MAPs to a participant. The high-dose group was included to maximise the chance of detecting an immune response, given that the population consisted of previously primed subjects, and this was the first Phase I study with MR HD-MAPs. The low dose was achieved by applying one MR-coated HD-MAP and two uncoated HD-MAPs to each participant. Participants in the placebo group received three uncoated HD-MAPs.

Participants were vaccinated on day 0. Application sites were selected to be free from scarring, tattoos, skin conditions, sunburn, and heavy hair. The area for application was marked and photographed. The foil seal on the HD-MAP was removed and the device was applied to the skin of the upper arm overlying the deltoid muscle. A slight pressure was applied to the top of the HD-MAP applicator device to activate the internal dome spring that propels the HD-MAP to the skin. The device was held in place for 60 s before being removed. All applications were performed by trained study team members.

Participants were monitored by clinical safety assessment visits on days 3, 7, 28, and 56, and phone calls on days 1 and 14. On day 0, all vaccination sites were assessed pre-vaccination and at 10 min, 1 h, and 2 h after HD-MAP or SC administration. Photographs of the treatment sites were taken at every clinical review. Skin reactions were assessed for erythema, swelling (further characterised as oedema or induration), tenderness, bruising, skin flaking, visibility, itching, and bleeding.

Three subjects dropped out of the study before the day-28 visit and were replaced with subjects randomly assigned to a treatment group. This resulted in the uneven group sizes shown in Figure 2.

### 2.5. Immunogenicity Assays

Serum blood samples were collected from participants on day 0 (pre-dose), 7, 28 and 56. Aliquots of serum were prepared using serum separation tubes and stored at −80 °C until analysis.

Analysis of measles and rubella serum IgG titres was carried out by Sullivan Nicolaides Pathology (Australia). For measles IgG, a chemiluminescence immunoassay was run using the Liaison KL instrument (measles IgG kit), and for rubella IgG, a two-step chemiluminescent microparticle immunoassay was run using the Abbott Architect i2000 instrument. The result for measles IgG (AU/mL) was negative if <13.50, equivocal if from 13.50 to 16.49, and positive if ≥16.50. The result for rubella IgG (IU/mL) was negative if <5.0, equivocal if 5.0 to 9.9, low positive if 10 to 20, and positive if ≥20. The definitions and cut-off thresholds used were as defined in the assays’ instructions.

A foci-reduction neutralization (FRN) assay was performed by 360biolabs (Australia) for each virus to measure functional antibodies against measles and rubella. Briefly, heat-inactivated human serum was titrated and incubated with a set concentration of measles or rubella virus and then inoculated onto Vero monolayers. An overlay medium of 0.5% carboxymethylcellulose in 2% FBS MEM was added to all wells, and the plates were incubated for 2 days at 37 °C or 5 days at 33 °C for measles and rubella, respectively. Plates were fixed with ice-cold acetone then immune-stained using anti-measles nucleoprotein mouse antibody (Abcam 106292) and anti-mouse IgG HRP (Abcam 97023) for measles, and anti-rubella capsid antibody (Abcam 34749) and anti-mouse IgG HRP (Abcam 97023) for rubella. TrueBlue Substrate was used to visualize foci, which were then counted. The neutralization titre is expressed as the reciprocal of the highest dilution at which 50% of foci formation is inhibited, as determined by interpolation of the 50% endpoint from the best-fit curve for each serum. The titre was converted to international units (IU) using the WHO 3rd International Standard for anti-measles serum (NIBSC 97/648) and WHO International Standard for anti-rubella serum (NIBSC RUBI-1-94).

Seroconversion for neutralizing antibodies was defined as an increase in antibody concentration from <0.120 IU/mL (for measles) [16] or <10 IU/mL (for rubella) [17] pre-vaccination, to concentrations above these values on day 28. For participants with antibody concentrations above these values at baseline, seroconversion was defined as a ≥4-fold increase in neutralizing antibody on day 28.

### 2.6. Thermostability

MR-coated HD-MAPs from the clinical batch were stored at 2–8 °C for various timepoints up to 24 months (study ongoing). At the initial (T0), 12-month, and 24-month timepoints, five HD-MAPs that had been stored at 2–8 °C were transferred to 40 °C for three days to mimic conditions required for controlled temperature chain (CTC) qualification (reviewed in [18]). Relative humidity (RH) for the 40 °C condition was 60% for T0 and 12 months and 75% RH for 24-month testing. At each timepoint, the dried vaccine coating was eluted from the HD-MAP microprojection tips and tested in the CCID_50_ potency assay. Prior to manufacture for the clinical trial, another thermostability study was conducted at 2–8 °C for various timepoints up to 30 months (study ongoing) and 25 ± 5 °C (60% RH) for 12 months. Accelerated testing of 3 days at 40 °C (60 ± 5% RH), 7 days at 37 °C (60 ± 5% RH) and 14 days at 37 °C (60 ± 5% RH) were also included. Lyophilized MR-Vac was also included in accelerated and long-term stability assays. Less frequent testing was performed for MR-Vac for long-term studies, and for all testing only two vials were sampled at each condition and timepoint. Therefore, the HD-MAP and MR-Vac thermostability data was not statistically compared. The CCID_50_ assay was performed using Vero (ATCC CCL-81) cells incubated for 6 days at 37 °C and RK13 (ATCC CCL-37) cells incubated for 10 days at 31 °C post-titration and inoculation of samples, for the detection of measles and rubella, respectively. Cytopathic effect was visually assessed after incubation and titre calculated using the Spearman–Karber method. Simple linear regression was performed to trend data, plotted with a 95% confidence band (GraphPad Prism 9.5.0).

### 2.7. Statistical Analyses (Immunogenicity)

For neutralizing antibodies, the two main analyses were two linear mixed regression models, one for measles and one for rubella, assessing for a change in log titre values from baseline. Categorical predictors were treatment group, visit (days 7, 28 and 56), and a group-by-visit interaction term. Baseline log titre value was also included as a continuous predictor. Titre values were modelled on the log-scale so that the model residuals were normally distributed. Visit-specific analysis of covariance (ANCOVAs) on day 7, 28 and 56 were performed to determine least square mean differences at each visit. These ANCOVAs assessed the same outcome as the linear mixed models, but only had two predictors: treatment group and log baseline antibody titre values. Models were fit using restricted maximum likelihood estimation, except for the likelihood ratio test models, which were generated using maximum likelihood estimation. A compound symmetry covariance structure was used. Analyses were performed in SAS version 9.4, and plots were prepared in GraphPad Prism version 9.5.0.

## 3. Results

### 3.1. Thermostability of MR HD-MAPs

**Figure 3:**
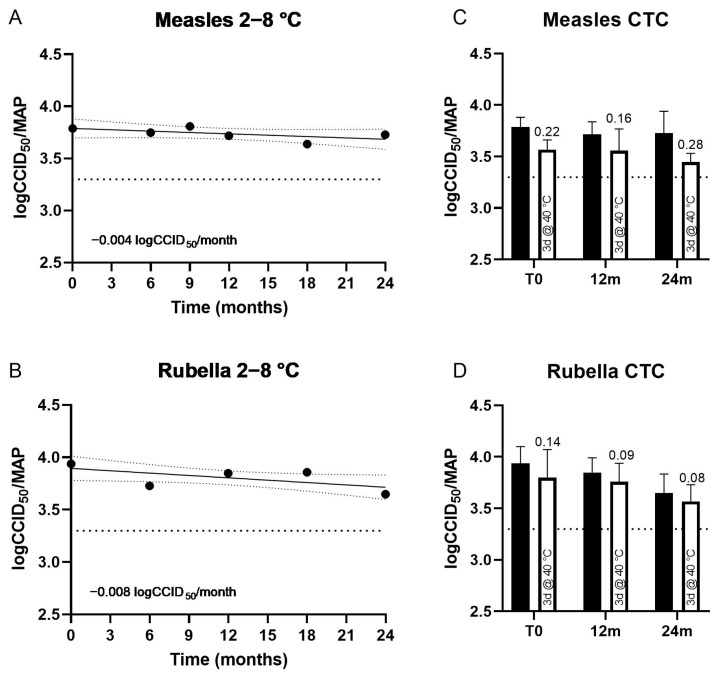
Long-term thermostability (potency) at 2–8 °C of measles (**A**) and rubella (**B**) on measles and rubella (MR) HD-MAPs used for clinical study. At nine months, rubella testing did not meet assay validity criteria, and an insufficient number of HD-MAPs were available to repeat testing. Linear regression was performed, and 95% confidence bands are shown. Stability of measles (**C**) and rubella (**D**) after 3 days at 40 °C following prior long-term storage at 2–8 °C for 12 months and 24 months (white bars), compared with HD-MAPs stored at 2–8 °C only (black bars). At the initial time point (T0), 12 months, and 24 months, five HD-MAPs were stored at 40 °C, 60–75% relative humidity (RH) for three days prior to testing (white bar), then assayed for measles (**C**) and rubella (**D**) potency in parallel with HD-MAPs stored at 2–8 °C (black bar). The average log loss relative to HD-MAPs stored at 2–8 °C and assayed in parallel is shown above the bar, bars represent 95% confidence interval. For all graphs, minimum specification (3.3 logCCID_50_ per virus per HD-MAP) is shown as a dotted line.

Real-time stability studies showed minimal loss of potency of M or R viruses, after storing MR HD-MAPs (clinical batch) at 2–8 °C for 24 months, with a degradation rate of 0.004 and 0.008 logCCID_50_ per MAP per month for measles and rubella, respectively (Figure 3A,C). Under CTC conditions of three days at 40 °C, 60%RH, MR HD-MAPs (clinical batch) showed minimal loss (up to 0.28 logCCID_50_/virus/MAP) compared to MAPs stored at 2–8 °C assayed in parallel, and still met minimum potency specifications (3.3 logCCID_50_/virus/MAP) under all temperature conditions at T0, 12, and 24 months (Figure 3B,D). Susceptibility to potency loss at 40 °C was similar for all conditions, and not influenced by the duration of prior storage at 2–8 °C.

In pre-clinical stability studies (Table 2), the rubella virus was more stable under all conditions in both MR-Vac and HD-MAPs, in comparison to the measles virus. MR HD-MAP stability for measles potency was improved on HD-MAPs, particularly for 14-day storage at 37 °C (60% RH). Stability of rubella was comparable between MR-Vac and HD-MAP. LogCCID_50_ loss was lower under some conditions for HD-MAPs compared to MR-Vac (7 days, 37 °C) but higher under others (30 months, 2–8 °C). For all conditions for rubella, only small losses were observed for both MR HD-MAPs and MR-Vac (up to 0.24 logCCID_50_).

At 24 months for the pre-clinical MR HD-MAPs and 6 months for the clinical MR HD-MAPs, other product attributes were also assessed, including sterility, applicator performance, and vaccine coating appearance. All testing met specifications set for product release into trials.

### 3.2. Particpants and Study Procedures

Between 9 July 2021 and 15 March 2022, 63 participants were enrolled into the study and vaccinated as described above (Figure 2). The original intention was to enrol only participants with detectable, but low titres of measles IgG. The trial was conducted during the COVID-19 pandemic and coincided with mass vaccination of the Australian population against SARS-CoV-2. Subjects were excluded if they had received a vaccine within 30 days of day 0 or planned on receiving a vaccine during the study period. This dramatically slowed recruitment rates. To complete the trial in a timely fashion, pre-screening, and enrolment on the basis of low baseline anti-measles serology was removed.

### 3.3. Summary of Adverse Events

All 63 subjects that received treatment were included in the safety analysis. There were no life-threatening or serious treatment-emergent adverse events (TEAEs), no TEAEs resulting in study withdrawal and no TEAEs resulting in death. TEAEs (28) deemed related to study treatment were experienced by 20 (31.7%) subjects, with 16 (25.4%) subjects experiencing 19 localized study treatment-related TEAEs and 7 (11.1%) subjects experiencing 9 systemic study treatment-related TEAEs. Treatment-emergent adverse events considered to be related to study treatment are listed in Table 3. Most adverse events were mild or moderate in severity, with only 1 (1.6%) subject in the active control group (MR-Vac) experiencing a severe TEAE (gastroenteritis).

### 3.4. Treatment Site Reactions and Resolution

Most HD-MAP application sites remained visible on day 7, with visibility noted in 48 (100.0%) sites for uncoated HD-MAP, 46 (95.8%) sites for low-dose HD-MAP and 48 (100.0%) sites for high-dose HD-MAP (Table 4). By day 28 and day 56, a minority of application sites were visible for uncoated and low-dose HD-MAP, while 46 (95%) and 26 (54.2%) of sites were visible in high-dose HD-MAP recipients on days 28 and 56, respectively. All application sites displayed a hyperpigmentation response and all application sites resolved. In contrast, for MR-Vac, 13 (86.7%) sites were visible at 10 min post-injection but by day 3, only 3 (20.0%) sites were visible. By day 7, none of the 15 injection sites were visible for the MR-Vac group (Table 3).

**Figure 4:**
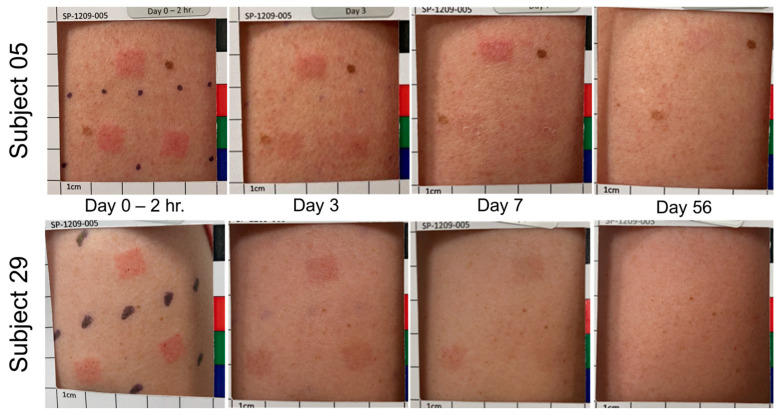
Application site appearance over time is shown for two subjects in the low-dose HD-MAP group. For Subject 05, the top application site was the MR HD-MAP. For Subject 29, the lower-left application site was the MR HD-MAP. Local responses shown were typical for the study.

Two representative examples of the appearance of the application site over time are shown in Figure 4. These participants were in the low-dose HD-MAP group and had two uncoated and one MR-coated HD-MAP applied. Low-dose participants are shown to demonstrate the difference in response and resolution between MR-coated and uncoated HD-MAPs. Participants in the high-dose MAP and uncoated MAP groups elicited similar responses to participants in the low-dose MAP group.

### 3.5. Serum Antibody Responses

**Figure 5:**
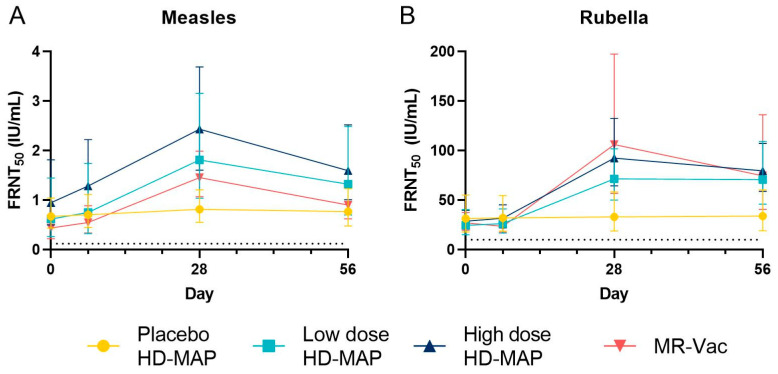
Neutralizing antibody concentrations for measles (**A**) and rubella (**B**). Serum was collected from subjects on day 0, 7, 28 and 56, and tested for neutralizing antibodies in the foci reduction neutralization (FRNT) assay, converted to International Units (IU) using WHO International Standard sera run in parallel. The geometric mean of the FRNT_50_ value (IU/mL) and 95% CI are shown for each group and day. The dotted line on the *y*-axis of each graph represents the protective threshold for each virus (0.120 IU/mL for measles, 10 IU/mL for rubella).

Nearly all participants (57 of 60 for both measles and rubella) had protective levels of measles and rubella neutralizing antibodies before vaccination (Figure 5 and Table 5).

There was no significant increase in FRN or ELISA titre and no seroconversions against measles or rubella in the uncoated MAP group after HD-MAP application (Figure 5, Table 4, and Supplementary Information). In contrast, FRN titres against measles and rubella significantly increased in all active groups following vaccination, peaking on day 28 post-vaccination. Titres declined slightly by day 56, but remained above baseline. At Day 28, titres for all active groups were significantly above placebo (*p* < 0.05) for both viruses, nor was there a statistically significant difference between MAP groups and MR Vac (*p* < 0.05) for both viruses. Seroconversion rates for neutralizing antibodies against measles on day 28 were 37.5%, 18.8% and 35.7% in the low-dose HD-MAP, high-dose HD-MAP, and MR-Vac groups, respectively. The corresponding seroconversion rates for rubella neutralizing antibodies were 37.5%, 25.0% and 35.7%.

**Figure 6:**
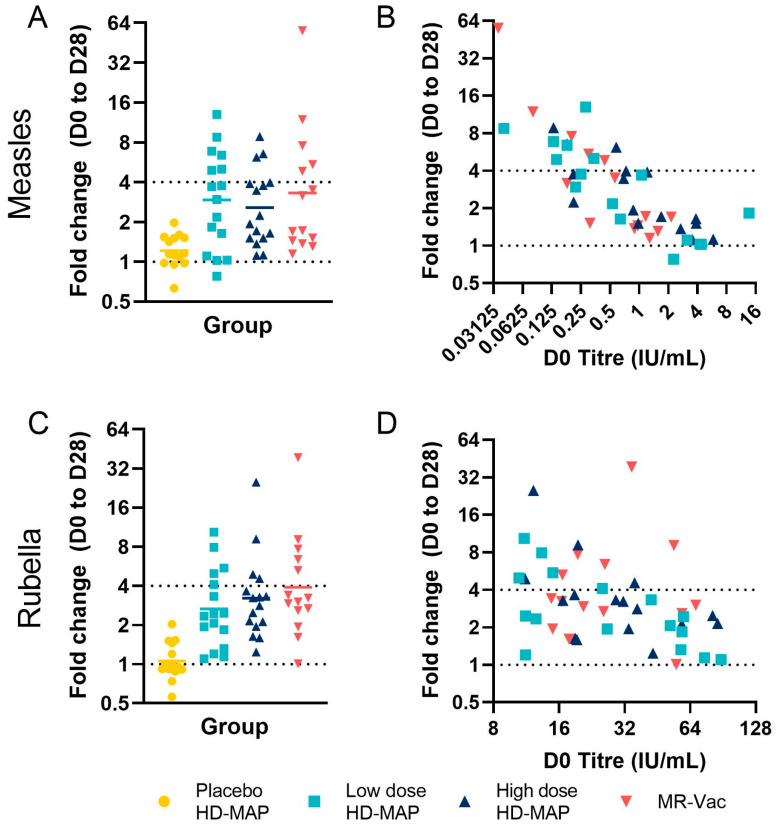
FRNT_50_ D0 to D28 fold change. (**A**) Measles and (**C**) rubella FRNT_50_ fold change between D0 and D28 for all groups; bar represents geometric mean. The relationship between D0 titre (*x* axis) and D28 fold change (*y* axis) is shown for (**B**) measles and (**D**) rubella. In both graphs, each point represents a single subject, coloured by group. Dotted lines represent seroconversion (fold change ≥ 4) or no change (fold change = 1).

The fold increase in titre, and therefore the seroconversion rates, were dependent on the pre-vaccination titre, with four-fold increases only being seen in participants with pre-vaccination titres ≤ 0.580 IU/mL for measles (Figure 6). For measles, there were nine, four, and eight such subjects in the low-dose, high-dose, and MR-Vac groups, respectively, including the six, three, and five subjects that seroconverted in each group, respectively (Figure 6A,B). The lower seroconversion rate to measles seen in the high-dose HD-MAP group in particular, could be attributed, at least in part, to the higher baseline anti-measles GMT in this group (Table 5). A similar pattern was seen for rubella. Across all groups, 15 subjects seroconverted for rubella antibodies on day 28; of these, 12 had pre-vaccination titres of ≤32 IU/mL (Figure 6C,D).

Anti-measles and anti-rubella IgG measured by ELISA showed a similar pattern of response to neutralizing antibodies (Table S1, Figure S1). Antibody concentrations peaked on day 28 and decreased slightly by day 56. As with neutralizing antibodies, the fold increase in titre was dependent on the baseline, pre-vaccination titre, with greater fold increases observed in participants with low IgG concentrations pre-vaccination.

## 4. Discussion

This Phase I trial was the first clinical trial of a live attenuated virus vaccine administered using the Vaxxas HD-MAP. The MR HD-MAP administration was well tolerated and induced immune responses similar to those achieved with SC injection. In addition, MR vaccines coated onto HD-MAPs were at least as thermostable as the standard, lyophilized vaccine.

The seroconversion rates to measles and rubella in this trial were relatively low, regardless of delivery method. This is most likely due to the participants having high antibody titres at baseline. Other Phase I trials of novel delivery devices for measles vaccines have also found that seroconversion rates were inversely correlated with baseline titre, and seroconversion rates were in the range of 7–17% for measles when participants with high starting titres were included [19,20]. A better indication of the immunogenicity of MR HD-MAPs will be provided from trials in naïve subjects.

Interestingly, an earlier study of transcutaneous (TC) delivery using skin abrasion followed by application of a projection-free MAP found that the TC delivery induced cell-mediated and mucosal immunity, but was a poor inducer of total or neutralizing antibody titres in the serum [21]. Cell-mediated and mucosal immunity were not measured in this trial, so it is not known whether MR HD-MAPs are strong inducers of these arms of the immune response, in addition to the serum antibody responses that were detected.

Delivery of subunit or inactivated vaccines by HD-MAPs has resulted in enhanced immunogenicity and/or dose-sparing in previous clinical trials [9,10]. Dose-sparing was not seen with either measles or rubella vaccines in this trial. The potency of the control SC vaccine was, however, lower than expected, so even if HD-MAPs did result in dose-sparing, it would not have been detected in this trial. It is also possible that ID or MAP delivery of measles and rubella, and of live attenuated vaccines in general, does not lead to dose-sparing. Dose-sparing has not been observed in several clinical trials using ID delivery of measles vaccine (reviewed in [22]).

The local reactogenicity seen with MR HD-MAPs was similar to that seen in a Phase I trial with an influenza vaccine, and might be at least in part due to the fact that recipients had been vaccinated previously with MR vaccines [10,23]. All reactions resolved completely. The local reactogenicity of MR HD-MAPs is a key issue that might affect acceptability. Therefore, reactogenicity will continue to be monitored in future clinical studies, which will be conducted in participants with darker skin pigmentation and in participants who are naïve to MR.

The study had several limitations. Other Phase I trials using novel devices to deliver a measles vaccine had pre-screened volunteers on the basis of anti-measles IgG titre, so that participants were seropositive, but had low baseline titres that allowed a booster response to be detected [20,21]. A retrospective analysis in one trial found an inverse correlation between baseline titre and detection of a booster response [19]. Our original plan to screen participants on the basis of anti-measles antibody titre had to be abandoned in this trial due to slow recruitment because of the COVID-19 pandemic. It is possible that less variability, more-potent immune responses, and a greater proportion of seroconversions would have been detected if we had enrolled only subjects with low concentrations of anti-measles IgG. Furthermore, as with most Phase I trials, the small group sizes limited the statistical power of the study to detect significant differences in immunogenicity between the different groups. A further limitation of the study was that it was conducted with adults in Australia and is not representative of the target populations for MR vaccine or MR HD-MAPs. Future clinical trials will be conducted in countries likely to use MR HD-MAPs if and when they are approved. The next clinical trial will be a Phase I/II trial conducted in The Gambia, and will include age de-escalation to include naïve infants. Future studies will also include at-risk populations, such as malnourished children.

MAPs as a platform have been prioritized by the Vaccine Innovation Prioritization Strategy (VIPs) as potentially transformational delivery devices that could address many of the key barriers to immunization in LMICs in general [13,24], and, in particular, those challenges facing MCVs [13]. Data are now being generated, including in this trial, to support this expectation. MR vaccines have been shown to have improved thermostability on other MAP formats compared with the standard, lyophilized presentation [25]. The controlled temperature excursion data presented here suggest that MR HD-MAPs will be suitable for use in the controlled temperature chain (CTC), facilitating their use in outreach settings. In addition, an end-user acceptability study in Nepal, Benin, and Vietnam found HD-MAPs to be highly acceptable for child immunization, and suggested that the technology should, once established, allow outreach delivery and administration by community health volunteers [26]. The simplicity and inherent safety of sharp-free HD-MAPs could allow for a broader pool of immunizers to reach the 15–20% of children that do not receive measles containing vaccines.

It is likely that MR HD-MAPs will have a higher cost of goods per dose than the current multi-dose, lyophilized presentations of MR vaccines. The cost premium may be mitigated by the improved thermostability of MR on HD-MAPs which should reduce the overloading required to compensate for loss of potency during the shelf-life of the vaccine, and vaccine wastage due to inadvertent heat exposure and wasted remaining doses in multi-dose vials. MR HD-MAPs could lead to further savings in the overall systems cost of MR immunization, by allowing lesser-trained personnel to administer vaccines, enabling CTC use which removes the need for cold-chain equipment in the ‘last mile’ of MR distribution, as well as broader health gains from improved coverage of MR vaccines. Therefore, an understanding of the potential impact of MR HD-MAPs on the total systems costs of MR vaccination is needed. An early study of the cost-effectiveness of MAPs for measles vaccination found that they could be cost-saving [27]. Now that more detailed information is available about the specific MR-MAP products being developed, value propositions of Full Vaccine Value Assessments for MR-MAPs are underway [28]. In addition, the possibility of including additional antigens, i.e., mumps and/or varicella, to MR-MAPs is being considered [29], as this might increase the commercial attractiveness of MCVs on MAPs. The additional antigens would, however, increase formulation and manufacturing complexity.

This trial was the first clinical trial using the Vaxxas HD-MAP as an integrated device, combining the MAP and the single-use applicator. It was also the first HD-MAP trial with a live attenuated vaccine. The positive data from the trial indicate that further development of MR HD-MAPs is warranted, and work is underway preparing for a Phase I/II age-de-escalation trial in adults and infants.

## Figures and Tables

**Table 1:** Baseline characteristics of participants (safety population).

Parameter	Value	Uncoated HD-MAP *n* = 16	Low-Dose HD-MAP *n* = 16	High-Dose HD-MAP *n* = 16	MR-Vac *n* = 15	Overall *n* = 63
Age (year)	*n*	16	16	16	15	63
	Mean (SD)	27.8 (7.4)	33.2 (9.9)	32.5 (9.3)	27.1 (10.3)	30.2 (9.4)
	Range	19–44	18–48	19–49	18–49	18–49
Sex, *n* (%)	Male	9 (56.3)	6 (37.5)	8 (50.0)	8 (53.3)	31 (49.2)
	Female	7 (43.8)	10 (62.5)	8 (50.0)	7 (46.7)	32 (50.8)
BMI (kg/m^2^)	*n*	16	16	16	15	63
	Mean (SD)	22.7 (2.096)	25.4 (3.7)	25.6 (3.8)	25.8 (3.914)	24.8 (3.6)
	Range	20.3–26.5	19.4–32.0	18.9–32.0	20.9–31.7	18.9–32.0
Race, *n* (%)	Aboriginal	0 (0.0)	0 (0.0)	0 (0.0)	1 (6.7)	1 (1.6)
	Asian	3 (18.8)	0 (0.0)	2 (12.5)	2 (13.3)	7 (11.1)
	Caucasian	12 (75.0)	14 (87.5)	14 (87.5)	12 (80.0)	52 (82.5)
	European and Filipino	0 (0.0)	1 (6.3)	0 (0.0)	0 (0.0)	1 (1.6)
	Hispanic	0 (0.0)	1 (6.3)	0 (0.0)	0 (0.0)	1 (1.6)
	Middle Eastern	1 (6.3)	0 (0.0)	0 (0.0)	0 (0.0)	1 (1.6)
Ethnicity, *n* (%)	Aboriginal/Torres Strait Islander	0 (0.0)	0 (0.0)	0 (0.0)	1 (6.7)	1 (1.6)
	Jewish	1 (6.3)	0 (0.0)	0 (0.0)	0 (0.0)	1 (1.6)
	Latin, Central and South American	0 (0.0)	1 (6.3)	0 (0.0)	0 (0.0)	1 (1.6)
	North-West European and Mediterranean	0 (0.0)	0 (0.0)	0 (0.0)	1 (6.7)	1 (1.6)
	North-East Asian	0 (0.0)	0 (0.0)	1 (6.3)	0 (0.0)	1 (1.6)
	North-West European	11 (68.8)	15 (93.8)	14 (87.5)	10 (66.7)	50 (79.4)
	South-East Asian	3 (18.8)	0 (0.0)	1 (6.3)	2 (13.3)	6 (9.5)
	Southern and Eastern European	1 (6.3)	0 (0.0)	0 (0.0)	1 (6.7)	2 (3.2)

**Table 2:** LogCCID_50_ loss for MR HD-MAPs and MR-Vac stored at 2–8 °C (pre-clinical studies).

Group	Time and Condition	LogCCID_50_ Loss (95% CI)			
		Measles		Rubella	
		MR HD-MAP	MR-Vac	MR HD-MAP	MR-Vac
Accelerated ^1^	3 days, 40 °C	0.39 (0.29–0.49)	0.37 (0.17–0.55)	0.16 (−0.06–0.38)	0.00 (−0.19–0.19)
	7 days, 37 °C	0.43 (0.33–0.53)	0.50 (0.31–0.69)	0.07 (−0.01–0.15)	0.33 (0.00–0.66)
	14 days, 37 °C	0.35 (0.26–0.44)	0.55 (0.55–0.55)	0.17 (−0.02–0.36)	0.05 (−0.35–0.45)
Long-term ^2^	6 months, 25 °C	0.67 (0.55–0.79)	0.75 (0.28–1.22)	0.24 (0.07–0.41)	0.23 (0.16–0.31)
	30 months, 2–8 °C	0.38 (0.21–0.55)	0.47 (0.35–0.59)	0.18 (0.13–0.23)	−0.03 (−0.25–0.19)
Long-term (clinical) ^2^	24 months, 2–8 °C	0.06 (−0.15–0.27)	Not carried out	0.29 (0.11–0.47)	Not carried out

Notes: ^1^—compared to 2–8 °C samples assayed in parallel; ^2^—compared to T0. All accelerated conditions were performed at 60 ± 5% RH. For reference, long-term data from the clinical batch are shown in Figure 3.

**Table 3:** Treatment-emergent adverse events considered to be related to study treatment.

	Low-Dose HD-MAP *n* = 16 *n* (%) [e]	High-Dose HD-MAP *n* = 16 *n* (%) [e]	Uncoated HD-MAP *n* = 16 *n* (%) [e]	MR-Vac *n* = 15 *n* (%) [e]
**Systemic**				
Fatigue	0	1 (6.3) [1]	0	0
Arthralgia	0	0	0	1 (6.7) [1]
Myalgia	0	0	0	1 (6.7) [1]
Headache	0	2 (12.5) [2]	2 (12.5) [2]	1 (6.7) [2]
**Local**				
Application site exfoliation	1 (6.3) [1]	0	1 (6.3) [1]	0
Injection site pain	1 (6.3) [1]	2 (12.5) [2]	1 (6.3) [1]	3 (20.0) [3]
Injection site pruritus	3 (18.8) [3]	6 (37.5) [7]	0	0

Note: For each AE, the results are presented as the number of subjects with the event, *n*; the proportion of subjects with the event, (%); and the number of events, [e].

**Table 4:** Resolution of treatment site reactions.

Parameter	Timepoint	Low-Dose HD-MAP *n* = 16	High-Dose HD-MAP *n* = 16	Uncoated HD-MAP *n* = 16	MR-Vac *n* = 15
Visible, No. (%)	Day 0 (10-Min PT)	48 (100.0)	48 (100.0)	48 (100.0)	13 (86.7)
	Day 0 (1-Hr PT)	48 (100.0)	48 (100.0)	48 (100.0)	11 (73.3)
	Day 0 (2-Hr PT)	48 (100.0)	48 (100.0)	48 (100.0)	11 (73.3)
	Day 3	48 (100.0)	48 (100.0)	45 (100.0)	3 (20.0)
	Day 7	46 (95.8)	48 (100.0)	48 (100.0)	0 (0.0)
	Day 28	16 (33.3)	46 (95.8)	10 (23.8)	0 (0.0)
	Day 56 or Early term.	7 (14.6)	26 (54.2)	3 (6.3)	0 (0.0)
Erythema, No. (%)	Day 0 (10-Min PT)	44 (91.7)	45 (93.8)	42 (87.5)	9 (60.0)
	Day 0 (1-Hr PT)	45 (93.8)	45 (93.8)	42 (87.5)	5 (33.3)
	Day 0 (2-Hr PT)	45 (93.8)	45 (93.8)	42 (87.5)	3 (20.0)
	Day 3	44 (91.7)	45 (93.8)	36 (80.0)	1 (6.7)
	Day 7	22 (45.8)	44 (91.7)	21 (43.8)	0 (0.0)
	Day 28	5 (10.4)	18 (37.5)	2 (4.8)	0 (0.0)
	Day 56 or Early term.	2 (4.2)	4 (8.3)	0 (0.0)	0 (0.0)
Swelling, No. (%)	Day 0 (10-Min PT)	42 (87.5)	39 (81.3)	41 (85.4)	7 (46.7)
	Day 0 (1-Hr PT)	42 (87.5)	42 (87.5)	39 (81.3)	1 (6.7)
	Day 0 (2-Hr PT)	43 (89.6)	41 (85.4)	33 (68.8)	0 (0.0)
	Day 3	8 (16.7)	31 (64.6)	0 (0.0)	0 (0.0)
	Day 7	10 (20.8)	32 (66.7)	0 (0.0)	0 (0.0)
	Day 28	1 (2.1)	0 (0.0)	0 (0.0)	0 (0.0)
	Day 56 or Early term.	0 (0.0)	0 (0.0)	0 (0.0)	0 (0.0)
Oedema, No. (%)	Day 0 (10-Min PT)	42 (87.5)	39 (81.3)	41 (85.4)	7 (46.7)
	Day 0 (1-Hr PT)	42 (87.5)	42 (87.5)	39 (81.3)	1 (6.7)
	Day 0 (2-Hr PT)	43 (89.6)	41 (85.4)	32 (66.7)	0 (0.0)
	Day 3	7 (14.6)	28 (58.3)	0 (0.0)	0 (0.0)
	Day 7	6 (12.5)	27 (56.3)	0 (0.0)	0 (0.0)
	Day 28 Day 56 or Early term.	0 (0.0) 0 (0.0)	0 (0.0) 0 (0.0)	0 (0.0) 0 (0.0)	0 (0.0) 0 (0.0)
Induration, No. (%)	Day 0 (10-Min PT)	0 (0.0)	0 (0.0)	0 (0.0)	0 (0.0)
	Day 0 (1-Hr PT)	0 (0.0)	0 (0.0)	0 (0.0)	0 (0.0)
	Day 0 (2-Hr PT)	0 (0.0)	0 (0.0)	1 (2.1)	0 (0.0)
	Day 3	1 (2.1)	3 (6.3)	0 (0.0)	0 (0.0)
	Day 7	4 (8.3)	5 (10.4)	0 (0.0)	0 (0.0)
	Day 28	1 (2.1)	0 (0.0)	0 (0.0)	0 (0.0)
	Day 56 or Early term.	0 (0.0)	0 (0.0)	0 (0.0)	0 (0.0)
Skin flaking, No. (%)	Day 0 (10-Min PT)	0 (0.0)	0 (0.0)	1 (2.1)	0 (0.0)
	Day 0 (1-Hr PT)	0 (0.0)	1 (2.1)	1 (2.1)	0 (0.0)
	Day 0 (2-Hr PT)	0 (0.0)	0 (0.0)	0 (0.0)	0 (0.0)
	Day 3	4 (8.3)	3 (6.3)	1 (2.2)	0 (0.0)
	Day 7	26 (54.2)	31 (64.6)	19 (39.6)	0 (0.0)
	Day 28	3 (6.3)	13 (27.1)	1 (2.4)	0 (0.0)
	Day 56 or Early term.	0 (0.0)	0 (0.0)	0 (0.0)	0 (0.0)

Note: The MR-Vac group had only one site per participant; other treatment groups had 3 sites per participants. Data were not collected for: 1 participant (3 application sites) in the uncoated HD-MAP group on day 3; 2 participants (6 application sites) in the uncoated HD-MAP group on day 28, and; 1 participant (1 injection site) in the MR-Vac group on day 28. The percentages for these data points are for the number of observations, not the total number per group.

**Table 5:** Measles and rubella neutralizing-antibody responses.

	Uncoated HD-MAP *n* = 14	Low-Dose HD-MAP *n* = 16	High-Dose HD-MAP *n* = 16	MR-Vac *n* = 14
**Measles**				
Day 0				
GMT IU/mL (95% CI)	0.673 (0.434–1.042)	0.617 (0.264–1.447)	0.949 (0.497–1.813)	0.439 (0.221–0.873)
Day 7				
GMT IU/mL (95% CI)	0.705 (0.447–1.113)	0.751 (0.323–1.742)	1.284 (0.742–2.222)	0.547 (0.337–0.889)
Seroconversion, No. (%)	0 (0)	0 (0)	1 (6.3)	2 (14.3)
Day 28				
GMT IU/mL (95% CI)	0.816 (0.55–1.209)	1.811 (1.039–3.156)	2.431 (1.603–3.687)	1.456 (1.067–1.987)
Seroconversion, No. (%)	0 (0)	6 (37.5)	3 (18.8)	5 (35.7)
Day 56				
GMT IU/mL (95% CI)	0.768 (0.478–1.233)	1.321 (0.702–2.484)	1.595 (1.01–2.518)	0.902 (0.623–1.306)
Seroconversion, No. (%)	0 (0)	3 (18.8)	1 (6.3)	3 (21.4)
**Rubella**				
Day 0				
GMT IU/mL (95% CI)	31.385 (17.875–55.104)	24.609 (15.005–40.358)	28.71 (20.697–39.826)	27.137 (19.651–37.475)
Day 7				
GMT IU/mL (95% CI)	32.025 (18.762–54.662)	26.214 (16.727–41.082)	31.675 (22.149–45.298)	23.527 (17.588–31.471)
Seroconversion, No. (%)	0 (0)	1 (6.3)	0 (0)	0 (0)
Day 28				
GMT IU/mL (95% CI)	33.164 (18.85–58.346)	71.38 (50.062–101.774)	92.385 (64.458–132.412)	106.053 (56.96–197.458)
Seroconversion, No. (%)	0 (0)	6 (37.5)	4 (25.0)	5 (35.7)
Day 56				
GMT IU/mL (95% CI)	33.894 (19.073–60.233)	70.821 (45.997–109.044)	79.572 (58.992–107.329)	74.518 (40.821–136.032)
Seroconversion, No. (%)	0 (0)	6 (37.5)	3 (18.8)	4 (28.6)

## Data Availability

Additional supporting data are available from the corresponding author upon reasonable request.

## Supplementary Materials

The following supporting information can be downloaded at: https://www.mdpi.com/article/10.3390/vaccines11111725/s1, Figure S1: ELISA responses; Table S1: Measles and rubella IgG responses.
