# Supplementary material for: Safety, Tolerability, and Immunogenicity of Measles and Rubella Vaccine Delivered with a High-Density Microarray Patch: Results from a Randomized, Partially Double-Blinded, Placebo-Controlled Phase I Clinical Trial
Source: Vaccines (Basel). 2023 Nov 17;11(11):1725. doi: 10.3390/vaccines11111725 (PMC10675090; doi:10.3390/vaccines11111725)
Supplement: Supplementary file 1 [file vaccines-11-01725-s001.zip › vaccines-2555606-supplementary.pdf]

Supplementary information

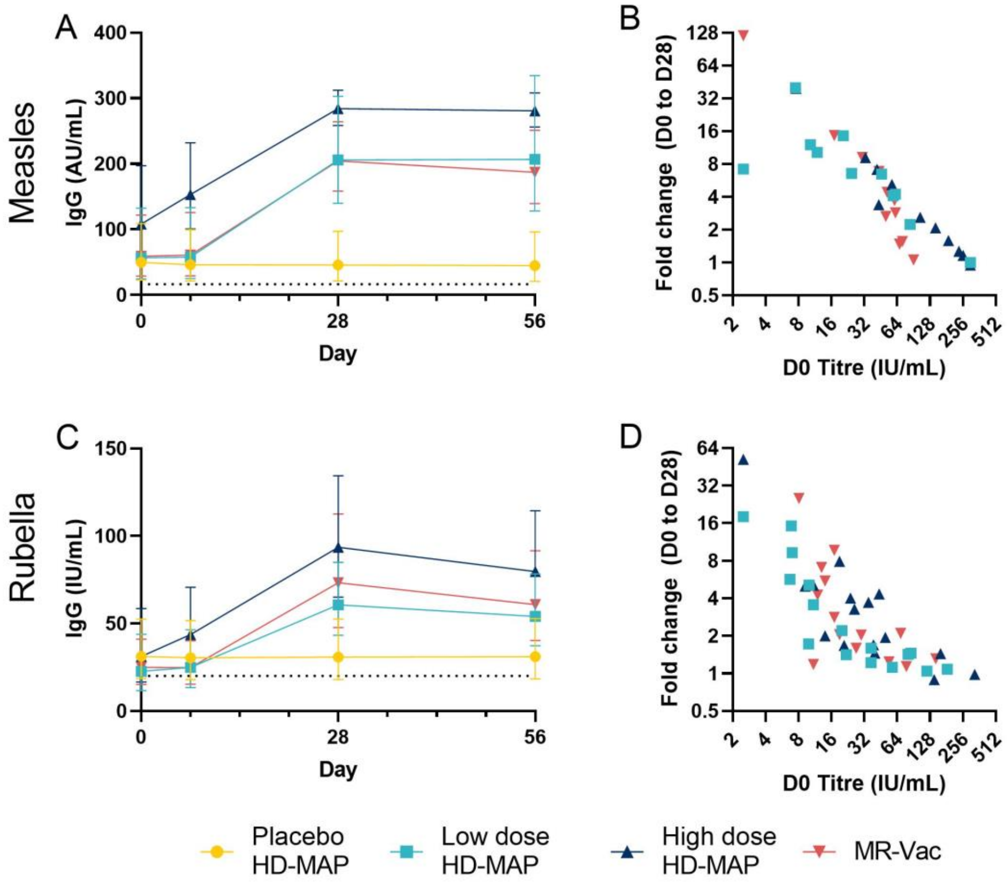

Figure S1. ELISA responses. IgG antibody concentrations for measles (A) and rubella (B). Serum was collected from subjects at Day 0, 7, 28, and 56 and IgG was measured using standard serological assays. The geometric mean of the IgG titer (AU/mL for measles, IU/mL for rubella) and 95% CI are shown for each group and day. The dotted line on the y-axis of each graph represents the positive threshold for each virus (16.5 IU/mL for measles, 20 IU/mL for rubella). The relationship between D0 titer (x-axis) and D28 fold change (y-axis) is shown for B) Measles and D) Rubella. In both graphs, each point represents a single subject, colored by group.

Supplementary Table S1. Measles and rubella IgG responses

	**Uncoated HD-MAP n=14**	**Low-dose HD-MAP n=16**	**High-dose HD-MAP n=16**	**MR-Vac n=14**
**Measles (AU/ml)**				
Day 0				
Geometric Mean Titer	49.675	56.877	108.318	59.309
(95% CI)	(22.704 – 108.69)	(24.418 - 132.484)	(59.474 - 197.275)	(28.837 - 121.982)
Positive, No. (%)	11 (78.6)	12 (75.0)	15 (93.8)	13 (92.9)
Day 7				
Geometric Mean Titer	46.155	57.764	153.138	60.718
(95% CI)	(21.437 - 99.377)	(25.038 - 133.261)	(100.939 - 232.33)	(29.331 - 125.69)
Positive, No. (%)	11 (78.6)	12 (75.0)	16 (100.00)	12 (85.7)

Day 28				
Geometric Mean Titer	45.67	205.783	284.125	204.619
(95% CI)	(21.447 - 97.249)	(139.796 - 302.917)	(258.661 - 312.094)	(158.45 - 264.241)
Positive, No. (%)	11 (78.6)	16 (100.0)	16 (100.00)	14 (100.00)
Day 56				
Geometric Mean Titer	44.711	206.974	280.885	187.143
(95% CI)	(20.757 - 96.306)	(128.06 - 334.515)	(256.152 - 308.007)	(139.473 - 251.105)
Positive, No. (%)	11 (78.6)	15 (93.8)	16 (100.00)	14 (100.00)
**Rubella (IU/ml)**				
Day 0				
Geometric Mean Titer	31.201	22.639	31.143	24.962
(95% CI)	(18.538 - 52.516)	(11.705 – 43.784)	(16.566 - 58.548)	(15.172 - 41.069)
Positive, No. (%)	12 (85.7)	11 (68.8)	14 (87.5)	13 (92.9)
Day 7				
Geometric Mean Titer	30.406	24.909	43.682	24.798
(95% CI)	(17.899 - 51.653)	(13.377 - 46.385)	(26.985 - 70.711)	(15.322 - 40.135)
Positive, No. (%)	12 (85.7)	12(75.00)	16 (100.00)	13 (92.9)
Day 28				
Geometric Mean Titer	30.845	60.635	93.552	73.255
(95% CI)	(18.08 - 52.622)	(43.307 - 84.895)	(65.116 - 134.406)	(47.658 - 112.601)
Positive, No. (%)	12 (85.7)	16 (100.00)	16 (100.00)	14 (100.00)
Day 56				
Geometric Mean Titer	31.112	54.030	79.631	60.762
(95% CI)	(18.33 - 52.805)	(37.272 - 78.32)	(55.39 - 114.481)	(40.326 - 91.555)
Positive, No. (%)	12 (85.7)	16 (100.00)	16 (100.00)	14 (100.00)
